# Editorial: Protein Export and Secretion Among Bacterial Pathogens

**DOI:** 10.3389/fcimb.2019.00473

**Published:** 2020-01-22

**Authors:** Thibault G. Sana, Romé Voulhoux, Denise M. Monack, Bérengère Ize, Sophie Bleves

**Affiliations:** ^1^Department of Microbiology and Immunology, Stanford School of Medicine, Stanford University, Stanford, CA, United States; ^2^Laboratoire d'Ingénierie des Systèmes Macromoléculaires, Institut de Microbiologie de la Méditerranée, CNRS, Aix-Marseille University, Marseille, France

**Keywords:** T2SS, T3SS, T6SS, T8SS, secretion systems, outer membrane proteins

Micro-organisms have colonized virtually every possible niche on Earth, including the bottom of the oceans and the upper atmosphere. Indeed, bacteria successfully evolved a myriad of complex systems to acclimate to, or to control, such different environments. In addition, bacterial pathogens face various environments within a host, including the microbiome and the immune system. In order to adapt to these harsh environments, pathogens often time utilize secretion systems. Secretion systems are machineries used to secrete proteins in the extracellular medium, or directly into the targeted cell.

Several secretion systems have been described in the scientific literature. Gram-negative bacteria have evolved eight secretion systems: T1SS (Type I Secretion System) to T6SS and the T9SS restricted so far to the phylum *Bacteroidetes*. The transport across the envelope can be a two-step process, in which exoproteins are first exported into the periplasm through the Sec or Tat export machineries, then attached on the surface or released into the extracellular medium which corresponds to the secretion step (T2-, T5-, and T9SS). The T1-, T3-, T4-, and T6SSs facilitate a one-step secretion process across the cell envelope leading to delivery of the effector in the medium (T1SS) or into host cells (T3-, T4-, and T6SS). All these secretion machinery components are made of proteins. However, an additional mechanism of secretion has been described and involves membrane shuffling which leads to the release of outer membrane vesicles (OMVs) loaded with proteins. Pathogens also use outer membrane proteins (OMPs), that are translocated through the inner membrane and then inserted in the outer membrane, for full virulence. In this special issue focused on the role of bacterial secretion systems during infections of mammalian hosts, several original research papers and reviews explore different facets of secretion systems amongst bacterial pathogens, as well as the different tools available in the scientific community to study them.

Several excellent reviews highlight and discuss important advances in the field.

A first review discuss about *Burkholderia pseudomallei*, the causative agent of melioidosis, a disease that can result in rapid, fatal infections in humans and animals. The mortality rate of melioidosis can reach 40% despite appropriate antibiotic therapies. Furthermore, diagnosis of *B. pseudomallei* infection is difficult because it can lead to a vast array of non-specific clinical manifestations, it is therefore predicted to be vastly under-reported. The genome of *B. pseudomallei* encodes three independent T3SS, named T3SS-1 to -3. While T3SS-1 and -2 have enigmatic roles, T3SS-3 is well-characterized and is required for escape from the endosome. In their review, Vander Broek and Steven discuss the current knowledge of *B. pseudomallei* T3SSs in the context of other well-characterized T3SSs.

Another bacterium highlighted in this issue, *Neisseria meningitidis*, can asymptotically reside in the nasopharynx of ~10% of the human population, but can cause meningococcal meningitis with high mortality rate. It secretes numerous proteins through a T1SS and various T5SSs, and also presents lipoproteins to its surface. These secretion systems and surface-exposed proteins are known to play a crucial role in *N. meningitidis* pathogenic interactions with the host. In their review, Tommassen and Arenas discuss the role of these secretion systems with a particular focus on the functions of secreted proteins.

Furthermore, two reviews describe the current knowledge on two different secretion systems. First, the T5SS comprise diverse branches, such as autotransporter and two-partner secretion (TPS). TPS system utilizes a partner transporter named TpsB for the secretion of an effector protein named TpsA. Historically, TPS systems have been shown to secrete large effector proteins involved in interactions between the bacterial pathogen and their host. Recently though, two novel roles in inter-bacterial competition and cooperation have been uncovered for these TPS. In their review, Guérin et al. discuss the latest discoveries regarding the translocation through TPS, and their roles. Then, the T9SS is a recently discovered secretion system present only in the phylum *Bacteroidetes*. It is composed of at least 18 proteins, but this secretion mechanism is still largely enigmatic. Interestingly, T9SS has two very distinct roles in environmental and pathogenic bacteria. While in environmental bacteria, it is involved in gliding motility, it is used as a weapon by pathogens. In their review, Lasica et al. present an up-to-date review on this intriguing secretion system.

Finally, three outstanding reviews describe and discuss the tools available to the scientific community to study secretion systems, and their cognate effectors. In the review by Gunasinghe et al., they explore how advances in fluorescent microscopy allowed direct imaging of the process of secretion. Indeed, using super-resolution microscopy, researchers can now image the dynamics, distribution, and translocation of secretion systems and effectors. Then, Lien and Lai review the methodologies that have been used to specifically identify T6SS effectors, as well as the function of their known effectors. They further propose strategies to identify potential new T6SS effectors. Finally, Maffei et al. provide an overview of the tools that have been developed to track protein secretion. Briefly, they review the biochemical, genetic, and imaging tools available to study secreted proteins and illustrate their respective advantages and limitations.

Following these reviews, several original papers are included in this issue that highlight new discoveries in the area of bacterial secretion systems. For example, they uncover novel functions of several effectors, and develop new tools to better study secretion systems in general. A first paper discuss about Enterotoxigenic *Bacteroides fragilis*, a human pathogen associated with childhood diarrhea and colon cancer. Its genome encodes the *B. fragilis* toxin (BFT), whose secretion leads to cleavage of cadherin, loss of cell adherence, and inflammation. However, this secretion mechanism was a mystery. In this issue, Zakharzhevskaya et al. showed that it is associated with Outer Membrane Vesicles (OMVs). A better understanding of BFT secretion will certainly provide new perspectives for prevention of *B. fragilis* infections in the future.

As discussed above, *Neisseria meningitidis* can cause meningococcal meningitis. It produces eight autotransporters, also called T5SSb, seven of which have been extensively studied. In their study, Arenas et al. revealed the role of the eighth autotransporter, AutB, of this bacterium. AutB, which is widely distributed among pathogenic *Neisseria* spp, is secreted and exposed to the cell surface, where it promotes biofilm formation and delays transcytosis *in vitro*. They further hypothesize that AutB could facilitate microcolony formation.

In another study, Hooda et al. describe a new analytical tool to identify surface lipoproteins (SLPs), which can be present or displayed in the outer membrane of Gram-negative bacteria. In *Neisseria*, the display of SLPs require surface lipoprotein assembly modulators (Slam). Using *in silico* analyses, Hooda et al. identified 832 Slam related sequences in 638 Gram-negative species, including several human pathogens. They further validated the surface display of one predicted Slam-adjacent protein in *Pasteurella multocida*, a zoonotic pathogen. Together, this suggests that SLPs and their interaction partner Slam are found widely in Proteobacteria.

Then, another paper discusses about *Acinetobacter baumanii*, a nosocomial human pathogen of high concern, because it rapidly acquires antibiotic resistance. In their study, Waack et al. provide evidence for a role of the T2SS of *A. baumanii* in protecting the bacteria from human complement. They further develop and optimize a simple high-throughput screen to identify small molecule inhibitors of the T2SS. This high-throughput screening was performed with 6,400 molecules, and could be performed on larger libraries to develop new *A. baumanii* therapeutics.

Finally, Meuskens et al. discuss about Outer Membrane Proteins (OMPs), which are important for adherence, protein secretion, biofilm formation, and virulence. However, high production of such proteins in a heterologous host for further characterization is usually challenging. In their study, the authors present a set of deletion mutants in *Escherichia coli* BL21 to specifically overproduce recombinant OMPs. The use of this engineered strain will facilitate future purification of OMPs. In addition, it will be useful for labeling experiments and biophysical measurements in the membrane environment.

Collectively, the reviews and original studies in this special issue explore a vast area of research, and describe new insights, new tools, and discusses several important aspects of protein secretion among bacterial pathogens.

## Author Contributions

TS and SB wrote the manuscript with contribution from RV, DM, and BI.

### Conflict of Interest

The authors declare that the research was conducted in the absence of any commercial or financial relationships that could be construed as a potential conflict of interest.

